# Identifying and Predicting Risk for Hospital Admission among Patients with Parkinsonism

**DOI:** 10.1002/mdc3.14257

**Published:** 2024-11-06

**Authors:** Emma Tenison, Anita McGrogan, Yoav Ben‐Shlomo, Emily J. Henderson

**Affiliations:** ^1^ Department of Population Health Sciences Bristol Medical School, University of Bristol Bristol United Kingdom; ^2^ Older People's Unit Royal United Hospitals Bath NHS Foundation Trust Bath United Kingdom; ^3^ Department of Life Sciences University of Bath, Claverton Down Bath United Kingdom; ^4^ The National Institute for Health and Care Research Applied Research Collaboration West (NIHR ARC West) at University Hospitals Bristol and Weston NHS Foundation Trust Bath United Kingdom

**Keywords:** emergency department, hospital admission, Parkinson's disease, parkinsonism, primary care database

## Abstract

**Background:**

Patients with parkinsonism are more likely than age‐matched controls to be admitted to hospital. It may be possible to reduce the cost and negative impact by identifying patients at highest risk and intervening to reduce hospital‐related costs. Predictive models have been developed in nonparkinsonism populations.

**Objectives:**

The aims were to (1) describe the reasons for admission, (2) describe the rates of hospital admission/emergency department attendance over time, and (3) use routine data to risk stratify unplanned hospital attendance in people with parkinsonism.

**Methods:**

This retrospective cohort study used Clinical Practice Research Datalink GOLD, a large UK primary care database, linked to hospital admission and emergency department attendance data. The primary diagnoses for nonelective admissions were categorized, and the frequencies were compared between parkinsonism cases and matched controls. Multilevel logistic and negative binomial regression models were used to estimate the risk of any and multiple admissions, respectively, for patients with parkinsonism.

**Results:**

There were 9189 patients with parkinsonism and 45,390 controls. The odds of emergency admission more than doubled from 2010 to 2019 (odds ratio [OR] 2.33; 95% confidence interval [CI] 1.96, 2.76; *P‐*value for trend <0.001). Pneumonia was the most common reason for admission among cases, followed by urinary tract infection. Increasing age, multimorbidity, parkinsonism duration, deprivation, and care home residence increased the odds of admission. Rural location was associated with reduced OR for admission (OR 0.79; 95% CI 0.70, 0.89).

**Conclusions:**

Our risk stratification tool may enable empirical targeting of interventions to reduce admission risk for parkinsonism patients.

Patients with parkinsonism, including the most common cause, idiopathic Parkinson's disease (PD), are proportionally more likely than age‐matched controls to have an emergency hospital admission,^1^ and this difference is most marked in younger individuals.^2^ A systematic review of emergency department (ED) attendances and hospital admissions in parkinsonism patients found that 16% to 45% visit ED once a year and 7% to 28% are admitted to hospital every year.^3^ Hospitalized patients may suffer a deterioration in motor symptoms or develop complications, such as infections or delirium, because of admission,^4^ and a proportion of patients are unable to return to their own home.^5^

The most common reasons for admission are falls, deterioration of motor and nonmotor symptoms of parkinsonism, cardiovascular events, and infections.^6^ Interventions that target so‐called “avoidable hospital admissions”^7^ may mitigate the significant financial cost^8^ and negative impact of hospitalization for parkinsonism patients.^4^ Risk modeling^7^, ^9^ offers a strategy to identify those at most risk where appropriate intervention might reduce the probability of hospital admission. Models to predict risk of unplanned hospital admission, such as the QAdmissions score,^10^ have been developed in nonparkinsonism populations. The limited studies in parkinsonism either excluded care home residents^11^ or were conducted in non‐UK tertiary centers,^12^ so may not be generalizable to people living with parkinsonism in the United Kingdom. Identifying predictors of hospital admission in a large, representative UK population of people with parkinsonism would help identify high‐risk subgroups to appropriately target interventions to reduce admission risk and/or reduce length of stay (LOS).

The objectives of this study were 3‐fold: to present (1) the reasons for hospital admission, (2) the rates of hospital admission and ED attendance over time, and (3) the adjusted predictors of unplanned hospital attendance to derive a risk stratification matrix for 1‐year risk of hospitalization for people with parkinsonism.

## Patients and Methods

### Sources of Data

The Clinical Practice Research Datalink (CPRD) GOLD database^13^ is a large primary care database, including 4.4% of the UK population as of July 2023,^14^ which enables researchers to study real‐world data. These data were linked to the Hospital Episode Statistics (HES), Office for National Statistics (ONS) death registration, and Index of Multiple Deprivation—an ecological marker of socioeconomic status and a rural–urban classification (ONS) based on practice postcode (Table S1 summarizes key terms).

### Study Design

This was a retrospective cohort study to compare the risk of ED attendance and hospital admission, based on patient characteristics, with the aim of predicting the annual risk of hospital admission in people with parkinsonism based on simple routinely collected variables.

### Study Population

The study population consisted of all prevalent patients with parkinsonism aged ≥35 years at the time of parkinsonism diagnosis from practices with research quality (“up to standard”) data, between January 1, 2010, and December 31, 2019. Patients were selected for inclusion if they had a diagnosis of parkinsonism, including idiopathic PD and related conditions (Supplementary Methods) but excluding drug‐induced parkinsonism, in the CPRD clinical and/or referral files. A validation study by Hernan et al. confirmed the PD diagnosis in 90% of those with a PD diagnosis in CPRD, together with 2 or more prescriptions for PD treatment.^15^ In this study, individuals were not excluded based on medication, because this may not routinely be prescribed for atypical parkinsonian syndromes and some idiopathic PD patients may be unmedicated. Each patient with parkinsonism was matched by age (±2 years), gender, and practice with up to 5 patients without a parkinsonism diagnosis, to compare the reasons for hospital admission in people with and without parkinsonism (objective 1).

### Outcomes, Exposures, and Confounders

Our outcomes for objective 3 were (1) any ED attendance, (2) any unplanned hospital admission, and (3) unplanned hospital admission for pneumonia; urinary tract infection (UTI); and falls, fractures, and head injuries as these are more common for people with parkinsonism. CPRD does not collect a direct measure of parkinsonism severity, so we derived proxy measures by utilizing disease duration, count of PD medication classes, and care home status. Potential confounders were secular period, age, gender, multimorbidity score (including PD), area deprivation level, and rural–urban classification (Supplementary Methods). Comorbidity was scored using the Cambridge Multimorbidity Score (CMS) as the presence or absence of 37 conditions based on medical and/or product codes.^16^, ^17^

### Statistical Analysis

Each subject had an index and censor date (death, migration, end of study) (Supplementary Methods), so study follow‐up was up to a maximum of 10 years. The reasons for emergency admissions (objective 1) were restricted to those that were not for zero bed day (ZBD) admissions, because ZBDs have less of an impact clinically and financially. The primary diagnoses were categorized into common reasons for nonelective admissions in parkinsonism patients, using lists of International Classification of Diseases 10th Revision (ICD‐10) codes compiled by Low et al.^1^ (personal communication, Prof. Yoav Ben‐Shlomo, January 31, 2022), which were then updated and expanded (Table S2). Multilevel generalized linear models, accounting for potential clustering by patient and practice, were used to calculate odds ratios (OR) to compare the proportion of all admissions that were due to a specific cause in parkinsonism cases versus controls. As the ORs were calculated from only cases and controls who have been admitted, it should be regarded as a proportional OR analogous to proportional mortality ratios.^18^

ED attendance and hospital admissions (including ZBD admissions) for parkinsonism patients were classified based on calendar year to examine secular trends (objective 2). For objectives 2 and 3, multilevel models were used to calculate effect estimates comparing attendance or admissions (all‐cause or cause‐specific) by exposure status (eg, calendar year for objective 2) and to allow for the repeated nature of the outcome that is clustered within individuals and to determine any practice effects (Supplementary Methods). We used logistic regression for the binary outcome of admission (yes/no) in each calendar year to model the OR of admission (for all and specific causes) or ED attendance for each potential predictor in turn. Our multivariable models were informed by our causal directed acyclic graph (Fig. S1). Negative binomial regression was used (rather than a Poisson model due to overdispersion) for analyses for the number (count) of admissions or ED attendances as the outcome (0, 1, 2, 3, etc.). We tested a priori for exploratory interactions between duration of parkinsonism with age and multimorbidity and whether there was effect modification for sex/gender (eg, if risk associated with exposure differed for men and women). We used multilevel generalized linear models to generate the predicted probabilities (with 95% confidence intervals [CI]) of hospital admission stratified by gender, age group, parkinsonism duration (0–2, 2–5, 5+ years), and multimorbidity score (0/1/2 conditions, 3/4 conditions, 5+ conditions), and accounting for interactions that had been identified, to provide a simple risk prediction tool for people with parkinsonism.

## Results

### Study Sample

**FIG. 1:**
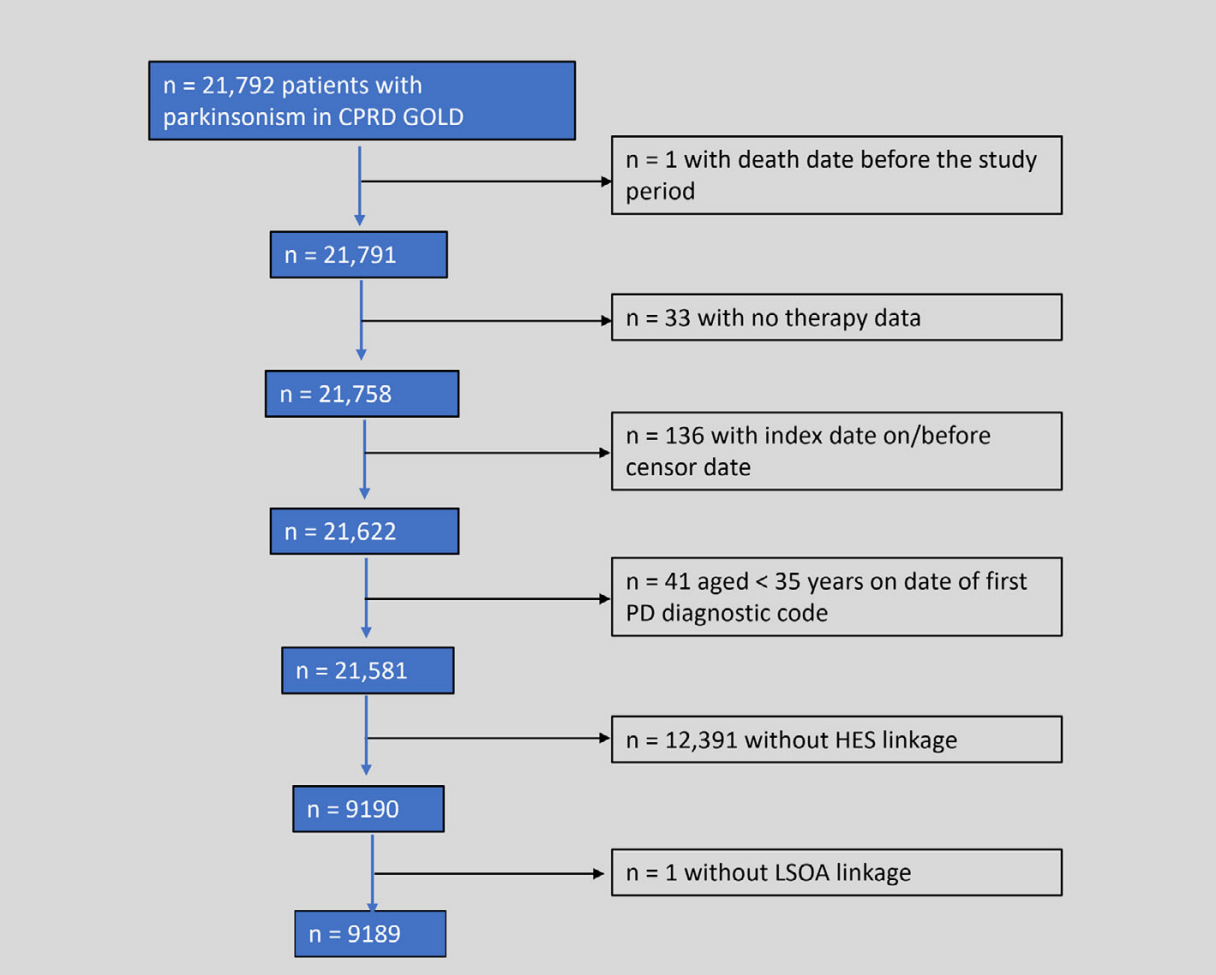
Flowchart of processing of the raw data for patients with parkinsonism to obtain the sample with linked data used in the analysis.

There were 21,792 patients with parkinsonism in the raw CPRD GOLD dataset. However, 12,391 had no linkage to HES, and 211 were excluded for other reasons (Fig. 1), leaving a sample of 9189 patients for analysis, with a median follow‐up of 2.3 years (interquartile range 1.0; 4.2), 59.5% male, 84.3% diagnosed with PD, mean age 77.1 (standard deviation 9.6) years, and median parkinsonism duration 2.5 years. There were 106,514 controls without parkinsonism in the raw CPRD GOLD dataset (included only in the analysis of objective 1). After data were cleaned and those without linkage were excluded, there were 45,390 patients for analysis (Fig. S2). Basic descriptive data for subjects with/without parkinsonism are presented in Table S3.

### Reasons for Emergency Hospital Admission in Cases versus Controls (Objective 1)

Among 9189 patients with parkinsonism and 45,390 patients without parkinsonism, 49.2% (4517) and 39.9% (18,106), respectively, had 1 or more non‐ZBD emergency admissions during the 10‐year study period.

For patients with parkinsonism 64.1% (6831) of the non‐ZBD emergency admissions could be classified into 1 of 9 system‐based categories (Table 1), and for patients without parkinsonism, 54.7% (24,447) were classified. The remaining emergency admissions consisted of multiple diagnostic codes each occurring at very low frequencies. Infection accounted for the greatest proportion of emergency admissions in cases and controls (23.6% and 19.2%, respectively). Falls and fractures accounted for 11.8% of emergency admissions in cases and 7.1% in controls.

**TABLE 1:** Emergency admissions, excluding zero bed day admissions, categorized into reasons and likely relationship to PD, in parkinsonism cases and controls

Categories	Relation to parkinsonism	Subcategories	n (% of all admissions)		Odds ratio* (95% CI)
			PD cases	Controls	
Parkinson's disease	Probably related	PD/dementia in PD	559 (5.2)	0^a^ (0.0)	N/A
Infections		UTI	910 (8.5)	2081 (4.7)	2.1 (1.9; 2.3)
		Pneumonia	1317 (12.4)	5151 (11.5)	1.2 (1.1; 1.3)
		Septicemia	182 (1.7)	751 (1.7)	1.1 (0.9; 1.3)
		Cellulitis	103 (1.0)	606 (1.4)	0.7 (0.6; 1.0)
Neuropsychiatric disorders		Old age without mention of psychosis	127 (1.2)	199 (0.5)	3.0 (2.3; 3.9)
		Disorientation	167 (1.6)	389 (0.9)	1.9 (1.5; 2.3)
		Delirium	68 (0.6)	154 (0.3)	1.9 (1.4; 2.6)
		Hallucinations	27 (0.3)	16 (0.0)	7.1 (3.8; 13.2)
Problems relating to blood pressure		Syncope and collapse	251 (2.4)	909 (2.0)	1.2 (1.0; 1.4)
		Orthostatic hypotension	147 (1.4)	237 (0.5)	2.7 (2.1; 3.5)
		Hypotension	17 (0.2)	54 (0.1)	1.3 (0.8; 2.3)
		Acute renal failure	107 (1.0)	621 (1.4)	0.7 (0.6; 0.9)
		Volume depletion	74 (0.7)	154 (0.3)	2.1 (1.5; 2.8)
Falls and fractures		Hip fracture	428 (4.0)	1190 (2.7)	1.6 (1.4; 1.9)
		Head injuries	220 (2.1)	558 (1.3)	1.7 (1.4; 2.0)
		Other fracture	179 (1.7)	700 (1.6)	1.1 (0.9; 1.3)
		Falls	434 (4.1)	701 (1.6)	3.0 (2.6; 3.5)
Gastrointestinal disorders		Constipation	146 (1.4)	360 (0.8)	1.7 (1.4; 2.1)
		Dysphagia	37 (0.4)	75 (0.2)	2.3 (1.5; 3.5)
	Unlikely to be related	Gastroenteritis	148 (1.4)	687 (1.5)	0.9 (0.7; 1.1)
		Nausea and vomiting	29 (0.3)	203 (0.5)	0.6 (0.4; 0.9)
		Upper GI bleed	102 (1.0)	681 (1.5)	0.6 (0.5; 0.8)
Respiratory		COPD	51 (0.1)	620 (1.4)	0.3 (0.2; 0.4)
		Asthma	18 (0.2)	134 (0.3)	0.6 (0.3; 1.1)
		Bronchiectasis	15 (0.1)	96 (0.2)	1.0 (0.3; 3.1)
Cardiovascular disorders		Cardiac related	598 (5.6)	4919 (11.0)	0.4 (0.4; 0.5)
		Stroke	127 (1.2)	1049 (2.4)	0.4 (0.4; 0.6)
		TIA	58 (0.5)	305 (0.7)	0.8 (0.5; 1.0)
Genitourinary and renal disorders		Urinary retention	86 (0.8)	375 (0.8)	1.0 (0.7; 1.3)
		Complications with catheter	48 (0.5)	141 (0.3)	1.5 (1.0; 2.2)
		Hematuria	53 (0.5)	313 (0.7)	0.7 (0.5; 1.0)
Other		All other admissions	3831 (35.9)	20,218 (45.3)	N/A

* Crude odds ratios were derived using a mixed‐effect generalized linear model.

^a^ Eighteen patients without parkinsonism (extracted based on an absence of a diagnostic code for parkinsonism in CPRD [Clinical Practice Research Datalink]) had an ICD‐10 code, indicating admission due to Parkinson's disease or Parkinson's disease dementia. This suggests a coding error in either the primary care (CPRD) or secondary care (Hospital Episode Statistics) record for these individuals or an error in the data extraction process.

Abbreviations: PD, Parkinson's disease; CI, confidence interval; UTI, urinary tract infection; GI, gastrointestinal; COPD, chronic obstructive pulmonary disease; N/A, not applicable; TIA, transient ischemic attack.

The majority (80.5%) of admissions for patients with parkinsonism were categorized as likely relating to parkinsonism (Table 1), with pneumonia being the most common precipitant in cases but with only slightly elevated odds compared to patients without parkinsonism (OR 1.2; 95% CI 1.1, 1.3). The next most common causes for parkinsonism patients were UTI, cardiac related, parkinsonism itself, and hip fracture. UTI was twice as common in patients with parkinsonism (OR 2.1; 95% CI 1.9, 2.3); admissions due to hip fracture were also more common (OR 1.6; 95% CI 1.4, 1.9). Less‐common reasons for admission, but likely related to parkinsonism, with larger ORs were hallucinations (OR 7.1; 95% CI 3.8, 13.2), falls (OR 3.0; 95% CI 2.6, 3.5), old age without mention of psychosis (OR 3.0; 95% CI 2.3, 3.9), orthostatic hypotension (OR 2.7; 95% CI 2.1, 3.5), dysphagia (OR 2.3, 95% CI 1.5, 3.5), constipation (OR 1.7; 95% CI 1.4, 2.1), and head injuries (OR 1.7; 95% CI 1.4, 2.0) (Table 1). Cardiac‐related admissions were less common in cases than controls, as were admissions due to stroke, transient ischemic attack, chronic obstructive pulmonary disease (COPD), acute renal failure, upper gastrointestinal bleed, and nausea/vomiting.

### Rates of Emergency Admission and ED Attendance over Time among People with Parkinsonism (Objective 2)

There were 12,647 emergency (including ZBD) admissions among parkinsonism patients during 25,938 person‐years of follow‐up. The mean rate of emergency admission was 0.49 per person‐year (95% CI 0.48, 0.50). There were 19,794 ED attendances, representing a mean ED attendance rate of 0.76 (95% CI 0.75, 0.77) per person‐year.

Emergency admission (0.40–0.60 per person‐year) and ED attendance (0.56–1.14 per person‐year) increased over the study period in a linear fashion; OR for 2019 versus 2010 was 2.33 (95% CI 1.96, 2.76) for emergency hospital admission and 2.31 (95% CI 1.98, 2.71) for ED attendance (Tables S4 and S5). The number of emergency admissions per person also increased linearly and was even larger (rate ratio 2019 vs. 2010; 2.85; 95% CI 2.45, 3.30). Similarly, the rate of ED attendance increased over 3‐fold (Rate ratio (RR) 3.25; 95% CI 2.88, 3.67).

### Demographic and Clinical Predictors for ED Attendance, Any Emergency Admission, and Specific Causes among People with Parkinsonism (Objective 3)

#### Simple Models, Adjusting Only for Study Year

Increasing age was associated with increased OR for ED attendance, all‐cause emergency admission (Table 2) and admission for pneumonia, UTI, and fall/fracture/head injury (Table 3). For example, patients aged 85 years compared to 35‐ to 64‐year‐olds had almost 6 times odds of emergency admission (OR 5.85; 95% CI 5.08, 6.74) and almost 9 times the odds of admission for fall/fracture/head injury (OR 8.76; 95% CI 6.24, 12.3), though this was less marked for ED attendance (OR 3.19; 95% CI 2.83, 3.61). Increasing multimorbidity score was associated with increased odds of ED attendance and emergency admission, including for all 3 presentations where the effect was strongest for pneumonia, followed by UTI.

**TABLE 2:** Odds ratios for predictors of emergency department attendance and all‐cause emergency hospital admission among patients with parkinsonism using logistic regression (simple model, adjusting only for study year)

	Emergency department attendance		Emergency hospital admission	
Predictor	Odds ratio; 95% CI	*P*‐value	Odds ratio; 95% CI	*P*‐value
Gender				
Male	Ref	0.036	Ref	0.90
Female	1.08 (1.00; 1.16)		1.01 (0.93; 1.09)	
Age (yr)				
35–64	Ref	<0.001*	Ref	<0.001*
65–69	1.09 (0.97; 1.24)		1.42 (1.23; 1.64)	
70–74	1.51 (1.34; 1.70)		2.07 (1.80; 2.37)	
75–79	2.06 (1.84; 2.30)		3.19 (2.79; 3.64)	
80–84	2.71 (2.42; 3.04)		4.53 (3.96; 5.17)	
85+	3.19 (2.83; 3.61)		5.85 (5.08; 6.74)	
Duration of parkinsonism (yr)				
<1	Ref	<0.001*	Ref	<0.001*
1–2.5	1.35 (1.24; 1.46)		1.28 (1.17; 1.40)	
2.5–5	1.51 (1.39; 1.64)		1.53 (1.40; 1.67)	
5–10	1.77 (1.62; 1.94)		1.71 (1.55; 1.89)	
10+	2.36 (2.11; 2.64)		2.18 (1.92; 2.48)	
Cambridge Multimorbidity Score				
0/1	Ref	<0.001*	Ref	<0.001*
2	1.37 (1.20; 1.57)		1.45 (1.23; 1.70)	
3	1.88 (1.65; 2.14)		2.08 (1.78; 2.43)	
4	2.42 (2.12; 2.77)		3.02 (2.59; 3.52)	
5	2.85 (2.49; 3.26)		3.59 (3.07; 4.20)	
6	3.57 (3.10; 4.12)		4.33 (3.68; 5.10)	
7+	4.27 (3.73; 4.90)		5.62 (4.80; 6.57)	
Care home status				
Not in a care home	Ref	0.058	Ref	0.002
Care home resident	1.15 (1.00; 1.34)		1.29 (1.10; 1.51)	
Count of PD medication classes				
0	Ref	<0.001*	Ref	0.001*
1	1.23 (1.14; 1.32)		1.15 (1.06; 1.25)	
2	1.29 (1.18; 1.42)		1.11 (1.00; 1.23)	
3	1.83 (1.61; 2.08)		1.39 (1.20; 1.60)	
4+	1.70 (1.37; 2.11)		1.10 (0.86; 1.42)	
Deprivation level				
1 (least deprived)	Ref	<0.001*	Ref	<0.001*
2	1.06 (0.93; 1.22)		1.07 (0.92; 1.24)	
3	1.10 (0.96; 1.26)		1.12 (0.96; 1.31)	
4	1.07 (0.93; 1.24)		1.12 (0.95; 1.31)	
5	1.37 (1.19; 1.58)		1.36 (1.17; 1.59)	
6	1.25 (1.07; 1.45)		1.30 (1.10; 1.53)	
7	1.39 (1.18; 1.63)		1.40 (1.17; 1.67)	
8	1.67 (1.42; 1.97)		1.72 (1.44; 2.05)	
9	1.60 (1.35; 1.90)		1.70 (1.41; 2.05)	
10 (most deprived)	1.75 (1.45; 2.10)		1.82 (1.49; 2.22)	
Rural–urban status				
Urban	Ref	<0.001	Ref	<0.001
Rural	0.73 (0.64; 0.83)		0.79 (0.70; 0.89)	

* *P*‐values for trend.

Abbreviations: CI, confidence interval; PD, Parkinson's disease; Ref, reference.

**TABLE 3:** Odds ratios for predictors of emergency admission due to pneumonia, urinary tract infection, and fall/fracture/head injury among patients with parkinsonism using logistic regression (simple model, adjusting only for study year)

	Emergency hospital admission for specific causes					
	Pneumonia		Urinary tract infection		Fall/fracture/head injury	
Predictor	Odds ratio; 95% CI	*P*‐value	Odds ratio; 95% CI	*P*‐value	Odds ratio; 95% CI	*P*‐value
Gender						
Male	Ref	<0.001	Ref	0.82	Ref	<0.001
Female	0.73 (0.63; 0.85)		1.02 (0.86; 1.21)		1.45 (1.28; 1.66)	
Age (yr)						
35–64	Ref	<0.001*	Ref	<0.001*	Ref	<0.001*
65–69	1.11 (0.78; 1.58)		1.87 (1.20; 2.91)		2.08 (1.41; 3.08)	
70–74	1.88 (1.37; 2.56)		2.99 (1.99; 4.49)		3.51 (2.47; 5.00)	
75–79	2.59 (1.92; 3.48)		4.93 (3.35; 7.25)		4.98 (3.55; 6.99)	
80–84	3.83 (2.85; 5.14)		6.18 (4.20; 9.09)		7.09 (5.07; 9.93)	
85+	6.45 (4.78; 8.71)		7.59 (5.12; 11.24)		8.76 (6.24; 12.31)	
Duration of parkinsonism (yr)						
<1	Ref	<0.001*	Ref	<0.001*	Ref	<0.001*
1–2.5	1.36 (1.12; 1.66)		1.42 (1.14; 1.77)		1.02 (0.85; 1.23)	
2.5–5	1.62 (1.33; 1.98)		1.58 (1.27; 1.96)		1.13 (0.94; 1.35)	
5–10	1.77 (1.43; 2.20)		1.68 (1.33; 2.12)		1.20 (0.99; 1.45)	
10+	1.85 (1.43; 2.41)		1.39 (1.03; 1.87)		1.78 (1.45; 2.19)	
Cambridge Multimorbidity Score						
		<0.001*		<0.001*		<0.001*
0/1	Ref		Ref		Ref	
2	1.63 (1.06; 2.51)		1.97 (1.21; 3.21)		1.17 (0.85; 1.61)	
3	2.63 (1.75; 3.96)		3.01 (1.89; 4.80)		1.51 (1.12; 2.06)	
4	3.87 (2.59; 5.77)		3.78 (2.38; 5.99)		1.87 (1.39; 2.53)	
5	4.83 (3.23; 7.22)		4.53 (2.85; 7.21)		2.03 (1.50; 2.75)	
6	6.42 (4.26; 9.67)		5.31 (3.30; 8.53)		2.52 (1.84; 3.44)	
7+	9.19 (6.19; 13.66)		7.07 (4.47; 11.19)		2.66 (1.97; 3.57)	
Care home status						
Not in a care home	Ref	<0.001	Ref	0.97	Ref	0.79
Care home resident	2.61 (2.01; 3.38)		0.99 (0.68; 1.44)		0.96 (0.70; 1.31)	
Count of PD medication classes						
0	Ref	0.42*	Ref	0.067*	Ref	0.39*
1	1.19 (1.01; 1.39)		1.20 (1.00; 1.44)		1.18 (1.02; 1.38)	
2	1.01 (0.82; 1.25)		0.74 (0.58; 0.96)		1.01 (0.83; 1.23)	
3	0.92 (0.67; 1.25)		0.87 (0.61; 1.25)		1.33 (1.03; 1.73)	
4+	0.71 (0.40; 1.28)		0.84 (0.45; 1.56)		0.89 (0.54; 1.47)	
Deprivation level						
1 (least deprived)	Ref	<0.001*	Ref	0.004*	Ref	0.081*
2	1.30 (0.98; 1.73)		1.15 (0.83; 1.59)		1.03 (0.80; 1.33)	
3	1.03 (0.76; 1.39)		1.09 (0.78; 1.52)		1.23 (0.96; 1.58)	
4	0.99 (0.72; 1.35)		1.14 (0.81; 1.61)		0.95 (0.72; 1.25)	
5	1.26 (0.93; 1.69)		1.14 (0.81; 1.58)		0.99 (0.76; 1.30)	
6	1.39 (1.02; 1.89)		1.23 (0.87; 1.75)		1.13 (0.86; 1.49)	
7	1.23 (0.87; 1.72)		1.08 (0.73; 1.60)		1.24 (0.92; 1.66)	
8	2.02 (1.47; 2.76)		1.23 (0.84; 1.80)		1.49 (1.12; 1.97)	
9	1.58 (1.12; 2.23)		1.80 (1.24; 2.62)		1.11 (0.80; 1.55)	
10 (most deprived)	2.08 (1.46; 2.96)		1.60 (1.06; 2.42)		1.09 (0.77; 1.55)	
Rural–urban status						
Urban	Ref	0.006	Ref	0.22	Ref	0.28
Rural	0.71 (0.56; 0.91)		0.85 (0.67; 1.10)		0.89 (0.73; 1.10)	

* *P*‐values for trend.

Abbreviations: CI, confidence interval; PD, Parkinson's disease; Ref, reference.

Female gender did not predict emergency admission overall but did predict increased admission for fall/fracture/head injury, a modest increase in ED attendance and reduced pneumonia admissions (Tables 2 and 3). Worsening deprivation level was associated with increased odds of emergency admission, including for pneumonia and UTI specifically. Registration with a rural practice was associated with 21% reduced odds of admission (OR 0.79; 95% CI 0.70, 0.89), a similar reduction in ED attendance and admission for pneumonia, but it did not predict admission for UTI or fall/fracture/head injury.

Duration of parkinsonism of >10 years was associated with a doubling of odds of admission (OR 2.18; 95% CI 1.92, 2.48) and ED attendance (OR 2.18; 95% CI 1.92, 2.48) compared to <1 year of parkinsonism (Table 2). Longer parkinsonism duration increased the odds of admission for pneumonia and UTI, as well as for fall/fracture/head injury but only after >10 years’ duration (Table 3). There was no clear association between increasing count of PD medication classes and emergency admission, though it did predict increased ED attendance. Care home residence was associated with elevated odds of hospital admission (OR 1.29; 95% CI 1.10, 1.51), whereas the effect on ED attendance was consistent with chance (*P* = 0.06) (Table 2). These individuals were over twice as likely to be admitted with pneumonia (OR 2.61; 95% CI 2.01, 3.38), but there was no association with admission for UTI or fall/fracture/head injury (Table 3). Similar patterns of results were obtained for the negative binomial regression models where the outcome was admission count (Table S6).

#### Adjusted Models

Adjustment for age did not alter the association between duration of parkinsonism and odds of ED attendance or emergency admission (all‐cause, for pneumonia or UTI), though there was a slight increase in the OR of admission for fall/fracture/head injury across all parkinsonism duration bands (Table S7). The reduced odds of ED attendance, all‐cause and pneumonia admission, for rural location were hardly altered by adjustment for age and deprivation (Table S8).

Care home residence was associated with a modest reduction in odds of admission (OR 0.84; 95% CI 0.72, 0.98) (Table 4), independent of age, parkinsonism duration, multimorbidity score, and gender, and a similar reduction in ED attendance (OR 0.80; 95% CI 0.69, 0.92). The increased odds of admission for pneumonia among care home residents remained but were attenuated after adjusting for potential confounders (Table 5). Where previously there was no association between care home status and admission for UTI or fall/fracture/head injury, after adjustment care home residence was associated with moderate evidence of reduced odds of admission for UTI (OR 0.62; 95% CI 0.43, 0.91) and strong evidence of reduced odds of fall/fracture/head injury (OR 0.60; 95% CI 0.44, 0.82) (Table 5).

**TABLE 4:** Odds ratios for the association between care home status and ED attendance and all‐cause emergency hospital admission among patients with parkinsonism, adjusting for study year in all cases and additionally for age, multimorbidity score, gender, and duration of parkinsonism [Correction added on 11 December 2024, after first online publication: In Table 4 the odds ratio (95% CI) and P‐value columns positioned under the column headings ‘Emergency department attendance’ and ‘Emergency hospital admission’ were reversed.]

Predictor/covariates	Emergency department attendance		Emergency hospital admission	
	Odd ratio (95% CI)	*P*‐value	Odds ratio (95% CI)	*P*‐value
Care home status	Ref 1.15 (1.00; 1.34)	0.058	Ref 1.29 (1.10; 1.51)	0.002
Care home status, adjusting for age	0.99 (0.85; 1.14)	0.87	1.03 (0.88; 1.20)	0.72
Care home status, adjusting for duration of parkinsonism	1.06 (0.91; 1.23)	0.46	1.18 (1.00; 1.38)	0.046
Care home status, adjusting for multimorbidity score	0.93 (0.80; 1.07)	0.32	1.01 (0.87; 1.18)	0.86
Care home status, adjusting for gender	1.15 (0.99; 1.33)	0.07	1.29 (1.10; 1.51)	0.002
Care home status, adjusting for age, duration of parkinsonism, multimorbidity score and gender	0.80 (0.69; 0.92)	0.002	0.84 (0.72; 0.98)	0.026

Abbreviations: ED, emergency department; CI, confidence interval; Ref, reference.

**TABLE 5:** Odds ratios for the association between care home status and emergency hospital admission for pneumonia, urinary tract infection, and fall/fracture/head injury, among patients with parkinsonism, adjusting for study year in all cases and additionally for age, multimorbidity score, gender, and duration of parkinsonism

	Reason for hospital admission					
Predictor/covariates	Pneumonia		UTI		Fall/fracture/head injury	
	*P*‐value	*P*‐value	OR (95% CI)	*P*‐value	OR (95% CI)	*P*‐value
Care home status	Ref	<0.001	Ref	0.97	Ref	0.79
	2.61 (2.01; 3.38)		0.99 (0.68; 1.44)		0.96 (0.70; 1.31)	
Care home status, adjusting for age	2.01 (1.55; 2.61)	<0.001	0.75 (0.52; 1.09)	0.13	0.71 (0.52; 0.96)	0.025
Care home status, adjusting for duration of parkinsonism	2.45 (1.87; 3.21)	<0.001	0.92 (0.63; 1.35)	0.67	0.89 (0.65; 1.22)	0.46
Care home status, adjusting for multimorbidity score	1.94 (1.50; 2.51)	<0.001	0.78 (0.53; 1.13)	0.19	0.81 (0.60; 1.11)	0.19
Care home status, adjusting for gender	2.70 (2.08; 3.50)	<0.001	0.99 (0.68; 1.44)	0.96	0.92 (0.67; 1.25)	0.59
Care home status, adjusting for age, duration of parkinsonism, multimorbidity score, and gender	1.67 (1.28; 2.17)	<0.001	0.62 (0.43; 0.91)	0.015	0.60 (0.44; 0.82)	0.001

Abbreviations: UTI, urinary tract infection; OR, odds ratio; CI, confidence interval; Ref, reference.

We found strong evidence for a negative interaction between parkinsonism duration and both age and multimorbidity score on the odds of emergency admission and ED attendance (Figs. S3–S8). Increasing disease duration predicted emergency admission and ED attendance, but the effect of disease duration was less marked in those in older age groups or with more comorbidities. Age and gender interacted so that, in older age groups, age had a greater effect on odds of ED attendance and admission (all‐cause and for pneumonia) in men than women (Figs. S9–S11). There was no evidence of an interaction between gender and either parkinsonism duration or multimorbidity score.

### Risk Stratification Matrix for Patients with Parkinsonism

Table 6 presents how the predictions generated by the multivariable model, including age group, parkinsonism duration, multimorbidity score, gender and study year (set to 2019), and accounting for interactions, can be presented for potential clinical use. The annual admission risk scores can be pragmatically classified into low (0%–15%), medium (16%–30%), and high (>30%). These cutoffs are arbitrary, though have face validity, and are similar to the QAdmissions thresholds.^10^, ^19^ The matrix shows how these 5 variables influence the risk of emergency admission so that in patients <70 years there were no high‐risk strata but, for men aged >85 years, only a CMS score of 0/1/2 and a duration <2 years were associated with a moderate risk, and the other 8 strata had high risk of admission.

**TABLE 6:** Risk‐prediction matrix illustrating how the 1‐year risk of emergency hospital admission predicted by the multivariable model (which included age group, duration of parkinsonism, gender, multimorbidity score, and study year, accounting for gender–age, duration–age, and duration–multimorbidity score interactions) can be presented for clinical use

	Male						Female				
Age	CMS↓	Parkinsonism duration→	<2 yr	2–4 yr	5+ yr	Age	CMS↓	Parkinsonism duration→	<2 yr	2–4 yr	5+ yr
35−64	0/1/2					35−64	0/1/2				
	3/4						3/4				
	5+						5+				
65−70	0/1/2					65−70	0/1/2				
	3/4						3/4				
	5+						5+				
70−74	0/1/2					70−74	0/1/2				
	3/4						3/4				
	5+						5+				
75−79	0/1/2					75−79	0/1/2				
	3/4						3/4				
	5+						5+				
80−84	0/1/2					80−84	0/1/2				
	3/4						3/4				
	5+						5+				
85+	0/1/2					85+	0/1/2				
	3/4						3/4				
	5+						5+				

	Annual risk 0%–15%
	Annual risk 16%–30%
	Annual risk >30%

Abbreviation: CMS, Cambridge Multimorbidity Score.

## Discussion

### Findings in the Context of the Existing Literature

Secular period, increasing age, multimorbidity score, duration of parkinsonism, and deprivation level, as well as care home residence and registration at a practice in an urban location, were associated with increased odds and rate of emergency hospital admission. Similar secular trends in emergency admissions have been noted in the general population and, because of an incomplete explanation by population growth or aging, this has been suggested to be due to factors such as increasing patient complexity, deprivation, and changing attitudes to risk.^20^, ^21^ There was no difference in odds of admission between men and women. Our findings mirror those reported by Okunoye et al.^2^ using routinely collected primary care data, who did not find a sex‐related difference in admission rates but reported that the rate of hospital admission increased with PD duration, though their incident cohort had a maximum PD duration of only 8 years,^2^ whereas our data allowed us to examine subjects with parkinsonism duration over 10 years.

An international multicenter study found that the higher number of comorbidities was a predictor of ED attendance and hospitalization,^12^ whereas another study, which included this cohort, found that age, previous hospital admission, and medication count predicted time to hospital encounter from baseline.^22^

There was modest evidence that care home residence reduced the odds of ED attendance and emergency admissions among parkinsonism patients after adjusting for potential confounders. This patient group is generally under‐researched, but a study of 90 patients in northeast England found that the median number of ED attendances, hospital admissions, and LOS was lower once living in a care home, compared to the period preceding institutionalization.^23^ Care home residents are likely to be more disabled and/or frail and therefore have advance care plans in place seeking to avoid admission. Our finding that this population is less likely to be admitted for fall/fracture/head injury suggests that continuous availability of care and monitoring can avoid the requirement for admission, which reflects head injury being recognized as an “additional nursing home–avoidable condition.”^24^ In keeping with other studies, care home residence increased the odds of admission for pneumonia,^23^ which likely reflects the need for treatments such as intravenous antibiotics and supplemental oxygen, usually delivered in hospital.

People with parkinsonism registered at a practice in a rural area had 18% lower odds of hospital admission and 24% lower odds of ED attendance. This novel finding has not been previously reported for this population, but a similar finding has been reported in patients admitted with asthma or COPD.^25^ It is hard to know how to interpret this finding; based on previous studies, this may be partly due to better management in the community or reduced access to secondary care^7^ as increasing distance from the ED has been associated with reduced emergency attendance.^25^, ^26^ Assuming these rural–urban differences are not explained by different clinical needs, it is unclear whether reduced health‐care contact results in better or worse longer‐term outcomes.

### Strengths and Limitations

There are several strengths to our study. CPRD uses a large, representative UK sample, and among the older population, almost everyone will be registered with a general practitioner. This will include patients across the spectrum of age, frailty, disease duration, and under‐researched groups such as care home residents, ethnic minority groups, and adults lacking the capacity to consent to research. We included patients at all stages of the disease course by not restricting to incident cases and included patients with all forms of parkinsonism, except drug‐induced parkinsonism, so our findings are more generalizable to clinical practice. Although the clinical course differs in atypical parkinsonian syndromes compared to idiopathic PD,^27^, ^28^ they share many of the same management challenges. Furthermore, there is often uncertainty surrounding the exact diagnosis, particularly in the early stages of the disease.^29^ We recognize the model may underestimate hospitalization risk when applied to people with atypical parkinsonism and slightly overestimate risk in those with idiopathic PD.

There was potential for diagnostic misclassification because data extraction of cases and controls relied on the presence or absence of diagnostic Read codes in the primary care files, rather than clinical confirmation. We found 18 people from 44,665 emergency admissions (4 per 10,000) without a code for parkinsonism but whose admission was coded as being for PD/PD dementia, suggesting a low error rate in either the primary care (CPRD) or hospital (HES) records. There is no direct measure of PD severity, such as Hoehn & Yahr staging^30^ or Movement Disorder Society‐Sponsored Revision of the Unified Parkinson's Disease Rating Scale (MDS‐UPDRS),^31^ in CPRD. We used available proxy markers of disease severity such as disease duration as an indicator of advanced parkinsonism^32^ and multiple classes of PD medication to manage motor symptoms and complications associated with disease progression.^33^ However, the number of medication classes will not always reflect severity: nonmotor complications may necessitate tapering or withdrawal of PD medication later in the disease course, whereas patients with an atypical parkinsonian syndrome are less likely to be on parkinsonism‐specific treatment.^34^ Our risk‐prediction tool was developed in a single dataset and therefore needs to be replicated and calibrated in an independent second dataset in both the United Kingdom and other high‐income countries to reduce the risk of model overfitting and enhance generalizability. The absolute risk of admission is likely to differ between different countries dependent on both hospital and community facilities resulting in different admission thresholds.

### Clinical and Research Implications

#### Risk Stratification Tool

Our findings provide further insights into the reasons people with parkinsonism are admitted to hospital, including a potentially valuable risk stratification tool that can be used to classify individuals into 3 simple risk strata, though our current cut points can be altered as required. This provides an empirical basis with which to target interventions aimed at reducing admission risk or, when unavoidable, reducing LOS, to individuals within a population at the highest risk of admission/ED attendance. It is important to consider risk of admission in both absolute and relative terms. The former may be more relevant for clinicians and patients in making individual choices and can lead to different decisions as well as being more comprehensible for health‐care planning.^35^ The risk‐prediction model presented here used routine primary care codes, so calculation could be automated, making it feasible to implement in primary care after further validation, followed by work to establish if it is useful in clinical practice. For risk stratification to have maximum utility, there is a need to develop interventions that successfully reduce the risk of admission and/or improve outcomes in those admitted and, having identified an individual as “high risk,” to develop a personalized care plan prioritizing interventions aiming to address their specific needs. For example, assessment of bone health and falls risk with appropriate intervention^36^ will target the higher risk of hip fracture seen in parkinsonism patients, whereas speech and language therapy input would be key for a patient with dysphagia. Using the model to estimate an individual's risk would allow follow‐up frequency to be tailored to risk but, importantly, also enable clinicians to discuss these risks with patients and caregivers. This could facilitate advance care planning conversations, including decisions around whether to admit to hospital and for which conditions, in those at high admission risk, helping to align care to patients' goals and priorities.^37^

#### Rural–Urban Differences

The reduced odds of admission and ED attendance in patients registered at a rural compared to urban practice, after adjusting for age and deprivation, may reflect a higher threshold among patients and general practitioners in rural areas to seek secondary care assessment, in part due to inaccessibility of health services. It is not clear, however, what threshold is optimum. The rural–urban differences may also reflect differing access to services or social support, which enable admissions to be avoided in one area but not another. Learning from these regional differences could inform the commissioning of services and better community support in urban areas.

Increasing age, multimorbidity score, and parkinsonism duration increased the odds of emergency admission, and there was an age–gender interaction. We have suggested how the model‐predicted risks can be presented for potential clinical use. Future research should seek to validate our risk‐prediction model and understand how risk is best communicated to different health‐care professionals, patients, and caregivers to shape implementation of this in clinical practice and maximize its utility.

## Author Roles

(1) Research project: A. Conception, B. Organization, C. Execution; (2) Statistical analysis: A. Design, B. Execution, C. Review and critique; (3) Manuscript preparation: A. Writing of the first draft, B. Review and critique.

E.T.: 1A, 1B, 1C, 2B, 3A

A.M.: 2C, 3B

Y.B.‐S.: 1A, 1B, 1C, 2A, 2B, 2C, 3B

E.J.H.: 1A, 1B, 1C, 2C, 3B

## Disclosures

**Ethical Compliance Statement**: This study is based on data from the Clinical Practice Research Datalink obtained under license from the UK Medicines and Healthcare Products Regulatory Agency (study protocol 20_000060). However, the interpretation and conclusions contained in this report are those of the authors alone. The authors confirm that the approval of an institutional review board and patient consent were not required for this work. They confirm that they have read the journal's position on issues involved in ethical publication and affirm that this work is consistent with those guidelines.

**Funding Sources and Conflicts of Interest**: This work was supported by Gatsby Charitable Foundation (GAT3676). Emma Tenison is funded by a National Institute for Health and Care Research Academic Clinical Lectureship. Emily J. Henderson is HEFCE funded by the University of Bristol for her academic work. Yoav Ben‐Shlomo is partly funded by the National Institute for Health and Care Research Applied Research Collaboration West (NIHR ARC West) and the University of Bristol. Anita McGrogan is HEFCE funded by the University of Bath. The authors declare that there are no conflicts of interest relevant to this work.

**Financial Disclosures for the Previous 12 Months**: Emily J. Henderson has received honoraria from the Neurology Academy and travel support from Bial. Yoav Ben‐Shlomo has received funding from Parkinson's UK, the Royal Osteoporosis Society, MRC, HQIP, the Templeton Foundation, Versus Arthritis, the Wellcome Trust, the National Institute of Health Research, and the Gatsby Foundation. Emma Tenison has received a speaker honorarium from the Neurology Academy. Anita McGrogan declares that that there are no additional disclosures to report.

## Supporting information


**Table S1.** Key terms used in the article and supplemental methods.
**Table S2.** ICD‐10 codes for each subcategory of admission.
**Table S3.** Characteristics of n = 9189 patients with parkinsonism and 45,390 patients without parkinsonism included in analysis.
**Table S4.** Emergency hospital admission and emergency department attendance rates by study year.
**Table S5.** Odds ratios by study year for emergency hospital admission and emergency department attendance, using logistic regression, and rate ratios for recurrent hospital admission and emergency department attendance, using negative binomial regression.
**Table S6.** Rate ratios for predictors of repeated emergency hospital admissions and ED (emergency department) attendance using negative binomial regression (simple model, adjusting only for study year).
**Table S7.** Odds ratios for the association between duration of parkinsonism (in years) and emergency admission (all‐cause and for specific causes) and emergency department attendance, adjusting for study year and additionally for age.
**Table S8.** Odds ratios for the association between rural–urban status and emergency admission (all‐cause and for specific causes) and ED (emergency department) attendance, adjusting for study year in all cases and additionally for age and deprivation.
**Figure S1.** Directed acyclic graph (DAG), developed using the DAGitty software to visually represent the potential causal and noncausal associations.
**Figure S2.** Flowchart of processing of the raw data for controls without parkinsonism to obtain the sample with linked data used in the analysis.
**Figure S3.** Predicted mean risk of emergency admission by age group in patients based on duration of parkinsonism (years); *P*‐value for likelihood ratio test = 0.002.
**Figure S4.** Predicted mean risk of emergency admission by multimorbidity score in patients based on duration of parkinsonism (years); *P*‐value for likelihood ratio test <0.001.
**Figure S5.** Predicted mean risk of emergency department attendance by duration of parkinsonism (years), based on age category (years); *P*‐value for likelihood ratio test <0.001.
**Figure S6.** Predicted mean risk of emergency department attendance by multimorbidity score in patients based on duration of parkinsonism (years); *P*‐value for likelihood ratio test <0.001.
**Figure S7.** Predicted mean risk of emergency admission for pneumonia by multimorbidity score in patients based on duration of parkinsonism (years); *P*‐value for likelihood ratio test = 0.029.
**Figure S8.** Predicted mean risk of admission for fall/fracture/head injury by age group in patients based on duration of parkinsonism (years); *P*‐value for likelihood ratio test <0.001.
**Figure S9.** Predicted mean risk of emergency department attendance by age group (years) in men and women; *P*‐value for likelihood ratio test = 0.001.
**Figure S10.** Predicted mean risk of emergency admission by age group (years) in men and women; *P*‐value for likelihood ratio test = 0.010.
**Figure S11.** Predicted mean risk of emergency admission for pneumonia by age group (years) in men and women; *P*‐value for likelihood ratio test = 0.010.

## Data Availability

This study is based on data from the Clinical Practice Research Datalink obtained under licence from the UK Medicines and Healthcare products Regulatory Agency (study protocol 20_000060). However, the interpretation and conclusions contained in this report are those of the author/s alone.
