# Supplementary material for: Identifying and Predicting Risk for Hospital Admission among Patients with Parkinsonism
Source: Mov Disord Clin Pract. 2024 Nov 6;12(1):43–56. doi: 10.1002/mdc3.14257 (PMC11736886; doi:10.1002/mdc3.14257)
Supplement: Supplementary file 1 — Table S1. Key terms used in the article and supplemental methods. Table S2. ICD‐10 codes for each subcategory of admission. Table S3. Characteristics of n = 9189 patients with parkinsonism and 45,390 patients without parkinsonism included in analysis. Table S4. Emergency hospital admission and emergency department attendance rates by study year. Table S5. Odds ratios by study year for emergency hospital admission and emergency department attendance, using logistic regression, and rate ratios for recurrent hospital admission and emergency department attendance, using negative binomial regression. Table S6. Rate ratios for predictors of repeated emergency hospital admissions and ED (emergency department) attendance using negative binomial regression (simple model, adjusting only for study year). Table S7. Odds ratios for the association between duration of parkinsonism (in years) and emergency admission (all‐cause and for specific causes) and emergency department attendance, adjusting for study year and additionally for age. Table S8. Odds ratios for the association between rural–urban status and emergency admission (all‐cause and for specific causes) and ED (emergency department) attendance, adjusting for study year in all cases and additionally for age and deprivation. Figure S1. Directed acyclic graph (DAG), developed using the DAGitty software to visually represent the potential causal and noncausal associations. Figure S2. Flowchart of processing of the raw data for controls without parkinsonism to obtain the sample with linked data used in the analysis. Figure S3. Predicted mean risk of emergency admission by age group in patients based on duration of parkinsonism (years); P‐value for likelihood ratio test = 0.002. Figure S4. Predicted mean risk of emergency admission by multimorbidity score in patients based on duration of parkinsonism (years); P‐value for likelihood ratio test <0.001. Figure S5. Predicted mean risk of emergency department attendance by duratio [file MDC3-12-43-s001.docx]

# SUPPLEMENTARY MATERIAL

## Supplementary methods

Table S1: key terms used in the manuscript and supplementary methods

| Term | Definition |
| --- | --- |
| Parkinsonism | A syndrome comprising bradykinesia, tremor, rigidity and postural instability, which occurs in idiopathic Parkinson’s Disease, but which can also be caused by other conditions such as progressive supranuclear palsy. This study included patients with all forms of parkinsonism except drug-induced parkinsonism. |
| Parkinson’s Disease (PD) | Used to refer to idiopathic Parkinson’s Disease, the most common cause of parkinsonism. |
| Deprivation level | An ecological marker of socioeconomic status. This study used the index of multiple deprivation data obtained at the patient level. |
| Practice | Used in this manuscript to refer to ‘general practices’, the organisations which provide primary care in the United Kingdom. Each practice contributing data to CPRD has a unique identifier. |
| Emergency Department (ED) attendance | A unique attendance (or visit) by an individual to an emergency department (also known as ‘accident and emergency’ or ‘A&E’), as recorded within Hospital Episode Statistics (HES) Accident and Emergency files. In some cases, an emergency department attendance may result in an emergency hospital admission. |
| Hospital admission, emergency hospital admission, unplanned hospital admission | A unique hospital ‘spell’ (which could comprise one or more episodes of care), which was classified within the Hospital Episode Statistics (HES) admitted patient care file as being for an ‘emergency’, as opposed to those categorised by HES as elective, maternity or ‘other’ admissions. These ‘emergency’ admissions may be triggered by referral from a variety of sources including the emergency department, general practitioner, or a consultant clinic. |

Table S2: ICD-10 codes for each sub-category of admission

| **Subcategories** | **Description** | **ICD-10 code** |
| --- | --- | --- |
| UTI | Urinary tract infection, site not specified | N390 |
| Pneumonia | Lobar pneumonia, unspecified | J181 |
|  | Unspecified acute lower respiratory infection | J22X |
|  | Pneumonia, unspecified | J189 |
|  | Pneumonitis due to food and vomit | J690 |
|  | Chronic obstruct pulmonary dis with acute lower resp infec | J440 |
|  | Bronchopneumonia, unspecified | J180 |
| Septicaemia | Sepsis, unspecified | A419 |
|  | Streptococcal sepsis | A40 |
|  | Other sepsis | A41 |
|  | Sepsis due to streptococcus, group A | A400 |
|  | Sepsis due to streptococcus, group B | A401 |
|  | Sepsis due to streptococcus, group D | A402 |
|  | Sepsis due to Streptococcus pneumoniae | A403 |
|  | Other streptococcal sepsis | A408 |
|  | Streptococcal sepsis, unspecified | A409 |
|  | Sepsis due to Staphylococcus aureus | A410 |
|  | Sepsis due to other specified staphylococcus | A411 |
|  | Sepsis due to unspecified staphylococcus | A412 |
|  | Sepsis due to Haemophilus influenzae | A413 |
|  | Sepsis due to anaerobes | A414 |
|  | Sepsis due to other Gram-negative organisms | A415 |
|  | Other specified sepsis | A418 |
| Cellulitis | Cellulitis of other parts of limb | L031 |
| Old age without mention of psychosis | Senility | R54X |
| Disorientation | Disorientation, unspecified | R410 |
| Delirium | Delirium, unspecified | F059 |
| Hallucinations | Hallucinations, unspecified | R443 |
|  | Visual hallucinations | R441 |
| Syncope and collapse | Syncope and collapse | R55X |
| Orthostatic hypotension | Orthostatic hypotension | I951 |
| Hypotension | Hypotension, unspecified | I959 |
| Acute kidney injury | Acute renal failure | N17 |
|  | Acute renal failure, unspecified | N179 |
|  | Other acute renal failure | N178 |
| Volume depletion | Volume depletion | E86X |
| Neck of femur fracture | Fracture of neck of femur | S720 |
|  | Peritrochanteric fracture | S721 |
|  | Subtrochanteric fracture | S722 |
| Head injuries | Unspecified injury of head | S099 |
|  | Superficial injury of other parts of head | S008 |
|  | Open wound of scalp | S010 |
|  | Superficial injury of head, part unspecified | S009 |
|  | Open wound of head, part unspecified | S019 |
|  | Superficial injury of scalp | S000 |
| Other fractures | Fracture of pubis | S325 |
|  | Fracture of lower end of radius | S525 |
|  | Fracture of upper end of humerus | S422 |
|  | Fracture of clavicle | S420 |
|  | Fracture of shaft of humerus | S423 |
|  | Multiple fractures of ribs | S224 |
|  | Fracture of lumbar vertebra | S320 |
|  | Fracture of lower end of both ulna and radius | S526 |
| Falls | Tendency to fall, not elsewhere classified | R296 |
|  | Unspecified fall | W19 |
|  | Unspecified fall- Home | W190 |
|  | Unspecified fall- Residential institution | W191 |
|  | Other fall from one level to another | W17 |
|  | Other fall from one level to another- Home | W170 |
|  | Other fall from one level to another- Residential institution | W171 |
|  | Fall on same level from slipping, tripping and stumbling | W01 |
|  | Fall on same level from slipping, tripping and stumbling- Home | W010 |
|  | Fall on same level from slipping, tripping and stumbling- Residential institution | W011 |
| Cardiac related | Chest pain, unspecified | R074 |
|  | Congestive heart failure | I500 |
|  | Atrial fibrillation and flutter | I48X |
|  | Other chest pain | R073 |
|  | Precordial pain | R072 |
|  | Unstable angina | I200 |
|  | Angina pectoris, unspecified | I209 |
|  | Acute myocardial infarction, unspecified | I219 |
|  | Left ventricular failure | I501 |
|  | Heart failure, unspecified | I509 |
|  | Atrial fibrillation and flutter | I48 |
|  | Acute transmural myocardial infarction of anterior wall | I210 |
|  | Acute transmural myocardial infarction of inferior wall | I211 |
|  | Acute transmural myocardial infarction of other sites | I212 |
|  | Acute transmural myocardial infarction of unspecified site | I213 |
|  | Acute subendocardial myocardial infarction | I214 |
|  | Acute myocardial infarction, unspecified | I219 |
|  | Atrioventricular block, complete | I442 |
| Stroke | Cerebral infarction, unspecified | I639 |
|  | Stroke, not specified as haemorrhage or infarction | I64X |
|  | Cerebral infarction due to unspecified occlusion or stenosis of cerebral arteries | I635 |
|  | Intracerebral haemorrhage, unspecified | I619 |
| TIA | Transient cerebral ischaemic attack, unspecified | G459 |
| Constipation | Constipation | K590 |
| Gastroenteritis | Noninfective gastroenteritis and colitis, unspecified | K529 |
|  | Enterocolitis due to Clostridium difficile | A047 |
|  | Viral intestinal infection, unspecified | A084 |
| Nausea and vomiting | Nausea and vomiting | R11X |
| Dysphagia | Dysphagia | R13X |
| GI bleed | Haematemesis | K920 |
|  | Melaena | K921 |
|  | Gastrointestinal haemorrhage, unspecified | K922 |
| Urinary retention | Retention of urine | R33X |
| Complications with catheter | Mechanical complication of urinary (indwelling) catheter | T830 |
|  | Fitting and adjustment of urinary device | Z466 |
| Haematuria | Unspecified haematuria | R31X |
| COPD | Chronic obstructive pulmonary disease with acute exacerbation, unspecified | J441 |
|  | Other specified chronic obstructive pulmonary disease | J448 |
|  | Chronic obstructive pulmonary disease, unspecified | J449 |
|  | Emphysema | J43 |
|  | Emphysema, unspecified | J439 |
|  | Other chronic obstructive pulmonary disease | J44 |
| Asthma | Asthma | J45 |
|  | Asthma, unspecified | J459 |
|  | Predominantly allergic asthma | J450 |
|  | Nonallergic asthma | J451 |
|  | Mixed asthma | J458 |
| Bronchiectasis | Bronchiectasis | J47X |
| PD as primary reason | Dementia in Parkinson disease | F023 |
|  | Parkinson disease | G20X |

Index of Multiple Deprivation (IMD) data were obtained at the patient level. This data is based on lower layer super output areas (LSOAs), which are clusters of adjacent postcode units with an average of 1,600 residents [1]. The IMD is a composite measure comprising several domains of material deprivation, including employment, crime, income, housing, living environment, education and skills, access to services and health. All national LSOAs are ranked from least deprived to most deprived and then divided into equal groups. For this study, we obtained IMD 2015 data categorised into deciles, where 1 represents the least deprived and 10 the most deprived.

Rural urban classification, categorised into a binary variable (rural/urban), was obtained at the practice level, based on the postcode of the GP practice, since CPRD will not provide both IMD and rural urban status at the patient level in order to avoid the small risk of reidentification. This classification is produced by the ONS, based on census population data, and assigns “rural” or “urban” status based on the percentage of the resident population living in these areas [2].

Patients were included if they had a diagnosis of parkinsonism, including idiopathic Parkinson’s disease, Parkinson’s disease dementia, atypical parkinsonian syndromes (multiple system atrophy, corticobasal degeneration, progressive supranuclear palsy, Lewy Body dementia), vascular parkinsonism, but excluding drug-induced parkinsonism.

Follow up of incident cases begun at parkinsonism diagnosis, or once under UTS follow-up and registered with the practice. Follow-up of prevalent cases begun on 1^st^ January 2010, or once under UTS follow-up and registered with the practice. The date at which the data contributed by a practice became UTS was obtained from the CPRD practice file and is derived by CPRD using an algorithm which determines the date at which practice data is research quality, based on gaps in the data and recording of deaths. The date at which a patient’s current registration with a practice began was obtained from the CPRD patient file.

The censor date, at which point the subject is no longer at risk, and no further events can be observed, was the earliest of last collection date for the practice (from the CPRD practice file), patient transfer out (from the CPRD patient file), death date or 31^st^ December 2019. The death date was taken to be the date of death recorded in the ONS death registration dataset, if available, or the date of death recorded in the CPRD patient file, since the former is recognised as the gold standard [3].

Likelihood ratio tests were used to determine whether it was necessary to account for clustering by GP practice, by comparing an empty model (without predictors) with and without the random intercept for GP practice. The intraclass correlation coefficient (ICC) was used to give an indication of the average correlation of observations for the same individual and of observations for patients at the same GP practice. Likelihood ratio tests to explore potential interactions based on a priori beliefs that duration of parkinsonism may interact with age and/or multimorbidity, and to determine if the effect of age, duration and number of comorbidities on risk of admission differed between men and women.


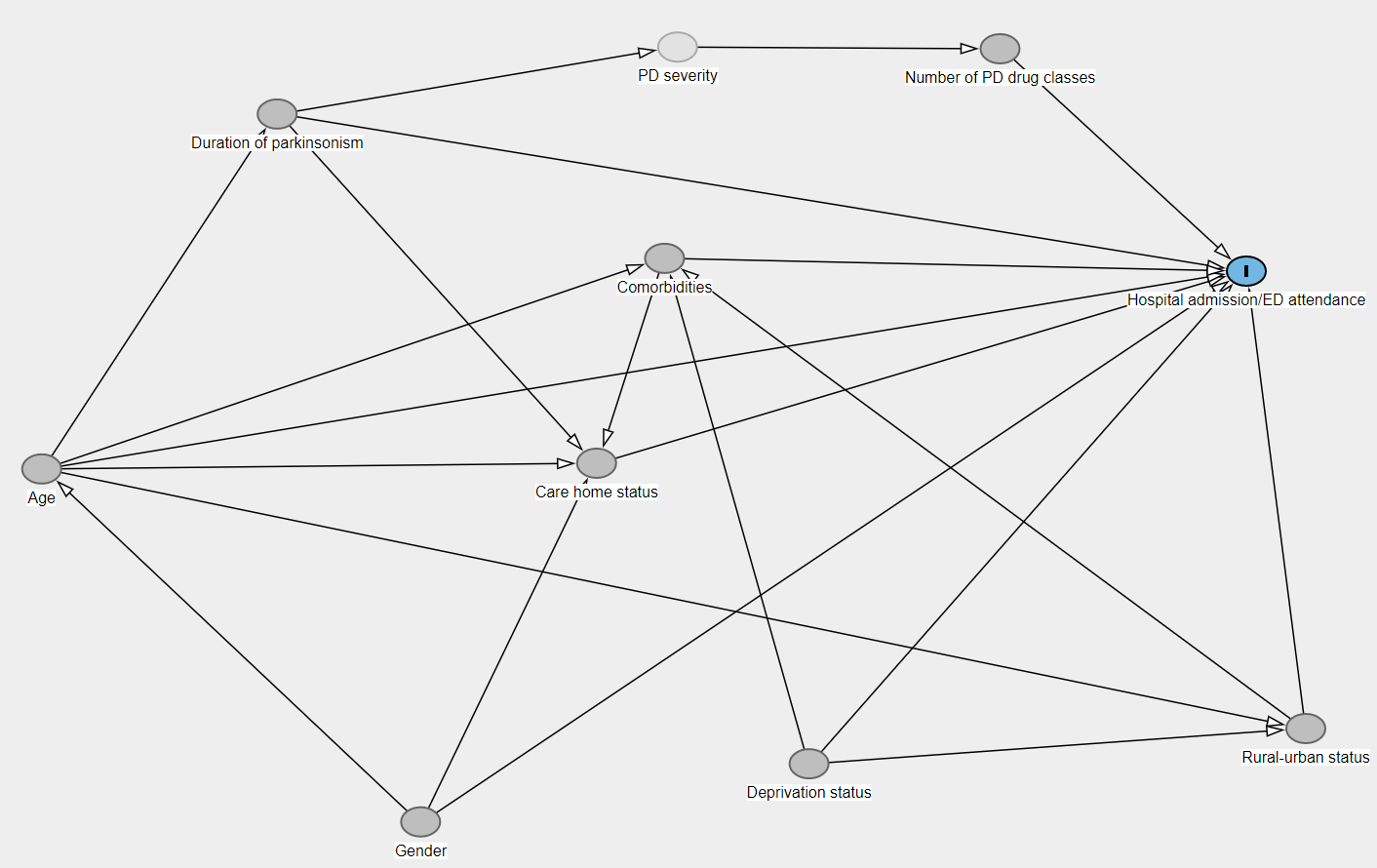


Figure S1: Directed acyclic graph (DAG), developed using the DAGitty software [4] in order to visually represent the potential causal and non-causal associations.

## Supplementary results


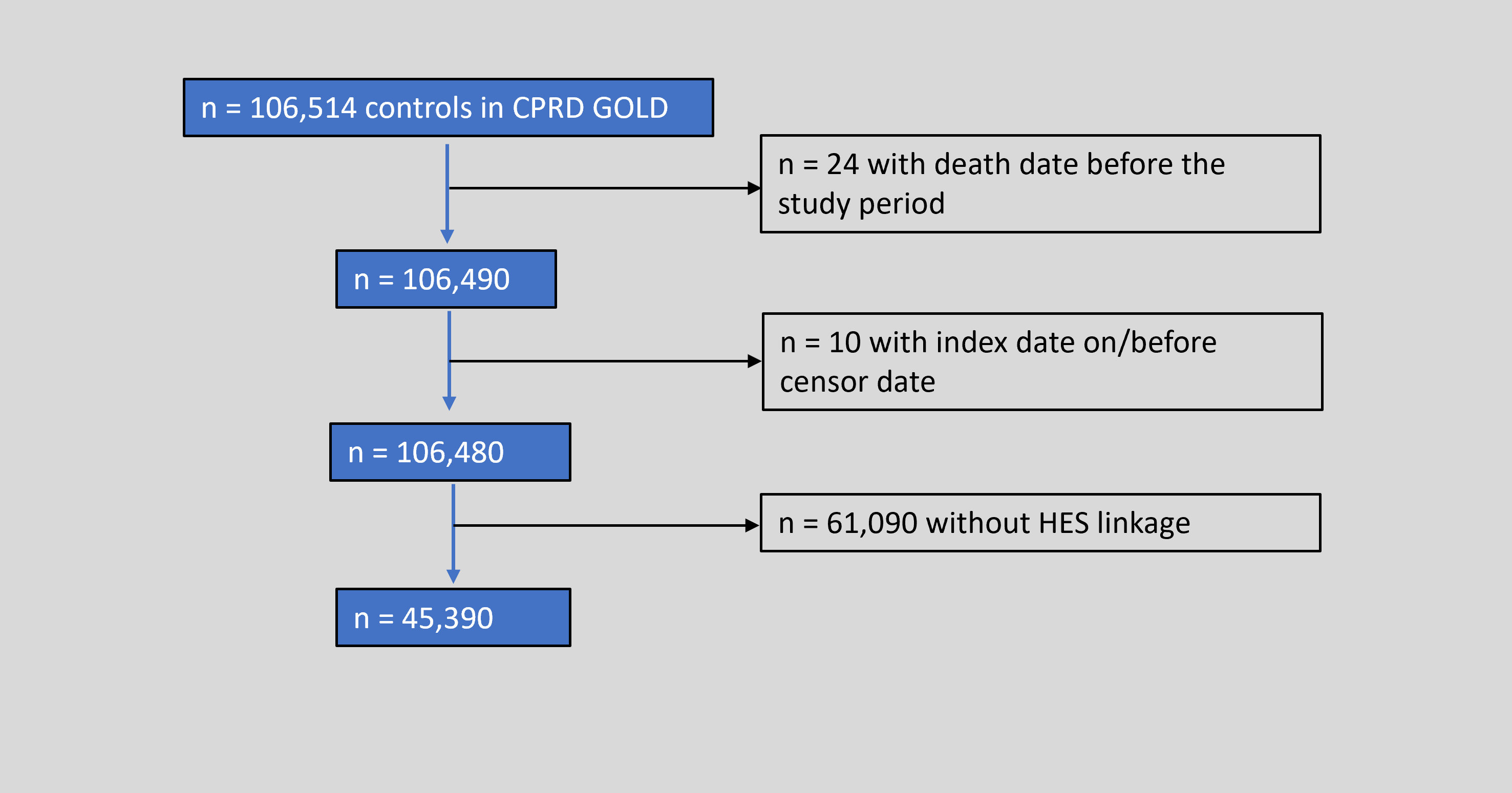


Figure S2: Flowchart of processing of the raw data for controls without parkinsonism to obtain the sample with linked data used in the analysis.

Table S3: Characteristics of n = 9189 patients with parkinsonism and 45,390 patients without parkinsonism included in analysis

| **Characteristic** | **Parkinsonism cases**  **n (%)** | **Non-parkinsonism controls**  **n (%)** |
| --- | --- | --- |
| Gender  Male  Female | 5,469 (59.5)  3,720 (40.5) | 26,934 (59.3)  18,456 (40.7) |
| Age (years)^&^  Mean (SD) | 77.1 (9.6) | 78.0 (9.8) |
| Duration of parkinsonism, based on date of first parkinsonism code (years)^&^  Median (IQR; range) | 2.5 (0.7- 5.8; 0- 39.5) | N/A |
| Type of parkinsonism first coded^$^  Parkinson’s disease  “Paralysis agitans”  Parkinsonism  Lewy Body Dementia  Progressive supranuclear palsy  Multiple system atrophy  Corticobasal degeneration  PD dementia  Cerebral degeneration in PD  Vascular parkinsonism | 7,747 (84.3)  121 (1.3)  4 (0.04)  703 (7.7)  172 (1.9)  64 (0.7)  21 (0.2) 79 (0.9)  2 (0.02) 276 (3.0) | N/A |
| Index of multiple deprivation score*  1 (least deprived)  2  3  4  5  6  7  8  9  10 (most deprived) | 1,317 (14.3) 1,213 (13.2)  1,120 (12.2)  991 (10.8) 1,192 (13.0) 905 (9.9)  699 (7.6) 705 (7.7)  561 (6.1)  479 (5.2) | 6,741 (14.9)  5,853 (12.9)  5,534 (12.2)  5,106 (11.3)  5,508 (12.1)  4,259 (9.4) 3,696 (8.2)  3,385 (7.5)  2,786 (6.1)  2,495 (5.5) |
| Rural-urban status (of GP practice)  Urban  Rural | 7,632 (83.1)  1,557 (16.9) | 37,649 (83.0)  7,741 (17.1) |
| ^&^At the start of the final year of study follow-up  ^$^As per the description of the medical term specified for each Read Code in the CPRD medical dictionary  *Of 45,390 non-parkinsonism controls, 27 do not have data available for IMD, hence the number of controls categorised as IMD levels 1 to 10 totals 45,363 (parkinsonism cases without LSOA linkage were dropped from analysis but non-parkinsonism controls without LSOA linkage were retained since IMD is not required for objective 1 which uses non-parkinsonism controls) | | |

Table S4: Emergency hospital admission and emergency department attendance rates by study year.

| **Year** | **Number of patients** | **Person years of follow up** | **Emergency admissions** | **Emergency admission rate per person year (95% CI)** | **Emergency department attendances** | **ED attendance rate per person year (95% CI)** |
| --- | --- | --- | --- | --- | --- | --- |
| 2010 | 3166 | 2,512 | 1015 | 0.40 (0.38; 0.43) | 1418 | 0.56 (0.54; 0.59) |
| 2011 | 3942 | 3,177 | 1481 | 0.47 (0.44; 0.49) | 2151 | 0.68 (0.65; 0.71) |
| 2012 | 4426 | 3,578 | 1621 | 0.45 (0.43; 0.48) | 2387 | 0.67 (0.64; 0.69) |
| 2013 | 4596 | 3,679 | 1709 | 0.46 (0.44; 0.49) | 2605 | 0.71 (0.68; 0.74) |
| 2014 | 4532 | 3,495 | 1768 | 0.51 (0.48; 0.53) | 2673 | 0.76 (0.74; 0.79) |
| 2015 | 3892 | 2,946 | 1508 | 0.51 (0.49; 0.54) | 2334 | 0.79 (0.76; 0.83) |
| 2016 | 2919 | 2,142 | 1086 | 0.51 (0.48; 0.54) | 1708 | 0.80 (0.76; 0.84) |
| 2017 | 2211 | 1,710 | 955 | 0.56 (0.52; 0.60) | 1513 | 0.88 (0.84; 0.93) |
| 2018 | 1852 | 1,457 | 763 | 0.52 (0.49; 0.56) | 1595 | 1.09 (1.04; 1.15) |
| 2019 | 1600 | 1,242 | 741 | 0.60 (0.55; 0.64) | 1410 | 1.14 (1.08; 1.20) |

Table S5: Odds ratios by study year for emergency hospital admission and emergency department attendance, using logistic regression, and rate ratios for recurrent hospital admission and emergency department attendance, using negative binomial regression.

| **Study year** | **Odds ratio (95% CI) for emergency hospital admission** | **Rate ratio (95% CI) for emergency hospital admission** | **Odds ratio (95% CI) for ED attendance** | **Rate ratio (95% CI) for ED attendance** |
| --- | --- | --- | --- | --- |
| 2010 | Reference | Reference | Reference | Reference |
| 2011 | 1.24 (1.09 to 1.41) | 1.22 (1.10; 1.36) | 1.29 (1.15; 1.45) | 1.24 (1.13; 1.36) |
| 2012 | 1.38 (1.22 to 1.57) | 1.33 (1.20; 1.48) | 1.44 (1.28; 1.61) | 1.33 (1.21; 1.46) |
| 2013 | 1.45 (1.28 to 1.65) | 1.50 (1.34; 1.67) | 1.57 (1.40; 1.77) | 1.50 (1.37; 1.64) |
| 2014 | 1.57 (1.38 to 1.78) | 1.74 (1.56; 1.95) | 1.68 (1.49; 1.88) | 1.74 (1.59; 1.92) |
| 2015 | 1.60 (1.40 to 1.83) | 1.85 (1.65; 2.07) | 1.69 (1.50; 1.91) | 1.86 (1.69; 2.05) |
| 2016 | 1.66 (1.44 to 1.92) | 1.99 (1.76; 2.26) | 1.75 (1.53; 1.99) | 2.01 (1.81; 2.24) |
| 2017 | 1.79 (1.53 to 2.10) | 2.32 (2.03; 2.65) | 1.93 (1.67; 2.22) | 2.33 (2.08; 2.61) |
| 2018 | 1.97 (1.67 to 2.32) | 2.34 (2.03; 2.70) | 2.14 (1.84; 2.48) | 2.92 (2.59; 3.28) |
| 2019 | 2.33 (1.96 to 2.76) | 2.85 (2.45; 3.30) | 2.31 (1.98; 2.71) | 3.25 (2.88; 3.67) |
| P-value for trend | <0.001 | < 0.001 | < 0.001 | < 0.001 |

Table S6: Rate ratios for predictors of repeated emergency hospital admissions and ED attendance using negative binomial regression (simple model, adjusting only for study year)

|  | **Emergency department attendance** | | **Emergency hospital admission** | |
| --- | --- | --- | --- | --- |
|  |  |  |  |  |
| **Predictor** | **Rate ratio;**  **95% CI** | **p-value** | **Rate ratio;**  **95% CI** | **p-value** |
| Gender  Male  Female | Ref  0.99 (0.92; 1.07) | 0.762 | Ref  1.04 (0.98; 1.11) | 0.206 |
| Age (years)  35-64  65-69  70-74  75- 79  80- 84  85+ | Ref  1.32 (1.16; 1.50)  2.03 (1.79; 2.30)  3.07 (2.72; 3.47)  4.40 (3.90; 4.98)  6.05 (5.32; 6.89) | < 0.001 | Ref  1.03 (0.93; 1.15)  1.45 (1.31; 1.61)  1.97 (1.79; 2.17)  2.61 (236; 2.88)  3.31 (2.97; 3.68) | < 0.001 |
| Duration of parkinsonism (years)  <1  1-2.5  2.5-5  5-10  10+ | Ref  1.03 (0.96; 1.11)  1.23 (1.13; 1.33)  1.43 (1.30; 1.56)  1.81 (1.61; 2.03) | < 0.001 | Ref  1.03 (0.96; 1.09)  1.16 (1.08; 1.23)  1.37 (1.27; 1.47)  1.74 (1.58; 1.92) | < 0.001 |
| Cambridge multimorbidity score  0/1  2  3  4  5  6  7+ | Ref  1.29 (1.12; 1.49)  1.87 (1.62; 2.15)  2.51 (2.19; 2.89)  3.06 (2.65; 3.52)  3.63 (3.13; 4.20)  4.58 (3.97; 5.29) | < 0.001 | Ref  1.23 (1.09; 1.38)  1.60 (1.42; 1.79)  1.98 (1.77; 2.22)  2.37 (2.11; 2.66)  2.78 (2.46; 3.13)  3.41 (3.03; 3.84) | < 0.001 |
| Care home status  Not in a care home  Care home resident | Ref  1.23 (1.06; 1.42) | 0.005 | Ref  1.10 (0.97; 1.25) | 0.130 |
| Count of PD medication classes  0  1  2  3  4+ | Ref  0.92 (0.86; 0.99)  0.85 (0.78; 0.94)  0.95 (0.84; 1.08)  0.87 (0.70; 1.08) | 0.017 | Ref  0.94 (0.88; 1.00)  0.95 (0.88; 1.02) 1.14 (1.03; 1.27)  1.12 (0.94; 1.34) | 0.163 |
| Deprivation level  1 (least deprived)  2  3  4  5  6  7  8  9  10 (most deprived) | Ref  1.09 (0.94; 1.26)  1.15 (0.99; 1.33)  1.10 (0.94; 1.28)  1.34 (1.15; 1.55)  1.33 (1.14; 1.55)  1.38 (1.6; 1.63)  1.74 (1.48; 2.06)  1.63 (1.36; 1.94)  1.87 (1.55; 2.26) | < 0.001 | Ref  1.08 (0.95; 1.22)  1.13 (0.99; 1.28)  1.05 (0.92; 1.20)  1.34 (1.17; 1.52)  1.25 (1.09; 1.44)  1.38 (1.19; 1.60)  1.64 (1.42; 1.90)  1.53 (1.31; 1.79)  1.79 (1.51; 2.11) | < 0.001 |
| Rural-urban status  Urban  Rural | Ref  0.77 (0.68; 0.87) | < 0.001 | Ref  0.72 (0.63; 0.81) | < 0.001 |

Table S7: Odds ratios for the association between duration of parkinsonism (in years) and emergency admission (all-cause and for specific causes) and emergency department attendance, adjusting for study year and additionally for age.

| **Predictor** | **Unadjusted** | **P-value for trend** | **Adjusting for age (years)** | **P-value for trend** |
| --- | --- | --- | --- | --- |
|  | **OR (95% CI);**  **p-value** |  | **OR (95% CI);**  **p-value** |  |
|  | For emergency hospital admission | | | |
| PD duration (years)  <1  1-2.5  2.5-5  5-10  10+ | Ref  1.28 (1.17; 1.40)  1.53 (1.40; 1.67)  1.71 (1.55; 1.89)  2.18 (1.92; 2.48) | < 0.001 | Ref  1.28 (1.17; 1.39)  1.50 (1.37; 1.64)  1.63 (1.48; 1.80)  2.03 (1.80; 2.29) | < 0.001 |
|  | For ED attendance | | | |
| PD duration (years)  <1  1-2.5  2.5-5  5-10  10+ | Ref  1.35 (1.24; 1.46)  1.51 (1.39; 1.64)  1.77 (1.62; 1.94)  2.36 (2.11; 2.64) | < 0.001 | Ref  1.35 (1.24; 1.46)  1.50 (1.38; 1.63)  1.73 (1.58; 1.89)  2.27 (2.03; 2.53) | < 0.001 |
|  | For pneumonia admission | | | |
| PD duration (years)  <1  1-2.5  2.5-5  5-10  10+ | Ref  1.36 (1.12; 1.66)  1.62 (1.33; 1.98)  1.77 (1.43; 2.20)  1.85 (1.43; 2.41) | < 0.001 | Ref  1.44 (1.18; 1.74)  1.73 (1.42; 2.11)  1.86 (1.50; 2.30)  1.88 (1.45; 2.43) | < 0.001 |
|  | For UTI admission | | | |
| PD duration (years)  <1  1-2.5  2.5-5  5-10  10+ | Ref  1.42 (1.14; 1.77)  1.58 (1.27; 1.96)  1.68 (1.33; 2.12)  1.39 (1.03; 1.87) | < 0.001 | Ref  1.46 (1.17; 1.81)  1.63 (1.31; 2.03)  1.71 (1.36; 2.16)  1.40 (1.04; 1.88) | < 0.001 |
|  | For falls/fracture/head injury admission | | | |
| PD duration (years)  <1  1-2.5  2.5-5  5-10  10+ | Ref  1.02 (0.85; 1.23)  1.13 (0.94; 1.35)  1.20 (0.99; 1.45)  1.78 (1.45; 2.19) | < 0.001 | Ref  1.05 (0.88; 1.27)  1.18 (0.98; 1.41)  1.24 (1.03; 1.50) 1.85 (1.51; 2.26) | < 0.001 |

Table S8: Odds ratios for the association between rural-urban status and emergency admission (all-cause and for specific causes) and ED attendance, adjusting for study year in all cases and additionally for age and deprivation.

| **Predictor/**  **covariates** | **Emergency department attendance** | | **Emergency hospital admission** | | **Reason for hospital admission** | | | | | |
| --- | --- | --- | --- | --- | --- | --- | --- | --- | --- | --- |
|  |  |  |  |  | **Pneumonia** | | **UTI** | | **Fall/fracture/ head injury** | |
|  | **Odd ratio**  **(95% CI)** | **p-value** | **Odds ratio**  **(95% CI)** | **95% CI** | **p-value** | **p-value** | **OR (95% CI)** | **p-value** | **OR (95% CI)** | **p-value** |
| Rural-urban status  Urban  Rural | Ref  0.79 (0.70; 0.89) | < 0.001 | Ref  0.73 (0.64; 0.83) | < 0.001 | Ref  0.71 (0.56; 0.91) | 0.006 | Ref  0.85 (0.67; 1.10) | 0.22 | 0.89 (0.73; 1.10) | 0.28 |
| Urban status, adjusting for age | 0.77 (0.68; 0.87) | < 0.001 | 0.72 (0.63; 0.81) | < 0.001 | 0.69 (0.55; 0.87) | 0.002 | 0.83 (0.65; 1.05) | 0.13 | 0.86 (0.70; 1.06) | 0.16 |
| Urban status, adjusting for deprivation | 0.84 (0.74; 0.95) | 0.005 | 0.77 (0.68; 0.88) | < 0.001 | 0.74 (0.59; 0.94) | 0.012 | 0.87 (0.68; 1.12) | 0.28 | 0.88 (0.71; 1.09) | 0.25 |
| Urban, adjusting for age and deprivation | 0.82 (0.73; 0.92) | 0.001 | 0.76 (0.67; 0.86) | < 0.001 | 0.73 (0.58; 0.93) | 0.009 | 0.86 (0.67; 1.10) | 0.22 | 0.87 (0.71; 1.08) | 0.20 |


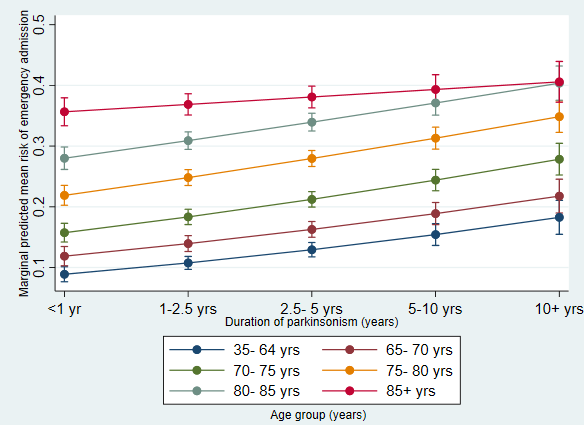


Figure S3: Predicted mean risk of emergency admission by age group in patients according to duration of parkinsonism (years; p-value for likelihood ratio test = 0.002.


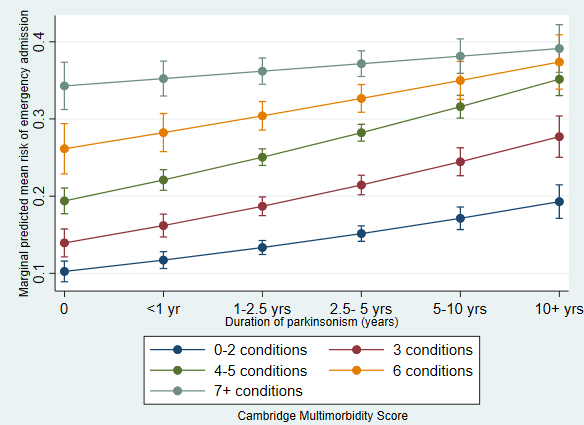


Figure S4: Predicted mean risk of emergency admission by multimorbidity score in patients according to duration of parkinsonism (years; p-value for likelihood ratio test < 0.001.


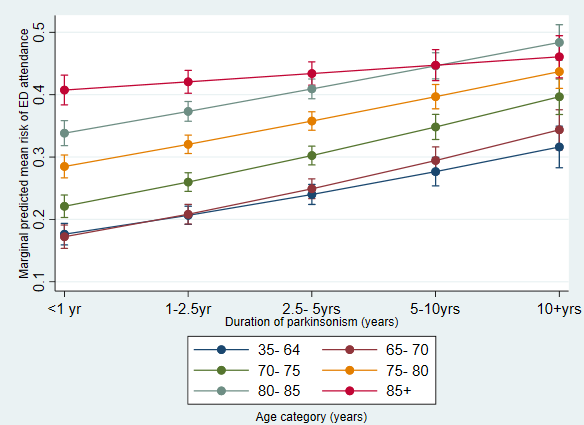


Figure S5: Predicted mean risk of emergency department attendance by duration of parkinsonism (years), according to age category (years); p-value for likelihood ratio test < 0.001.


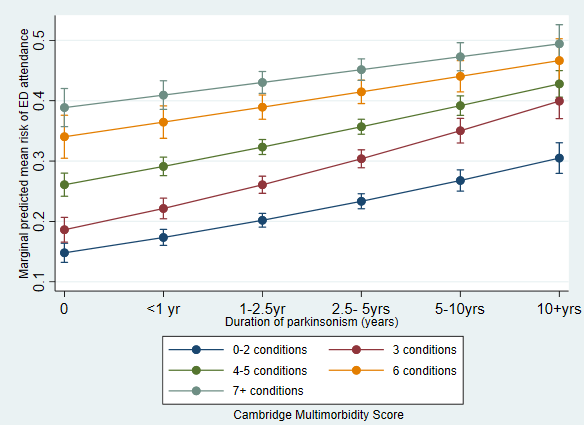


Figure S6: Predicted mean risk of emergency department attendance by multimorbidity score in patients according to duration of parkinsonism (years); p-value for likelihood ratio test < 0.001.


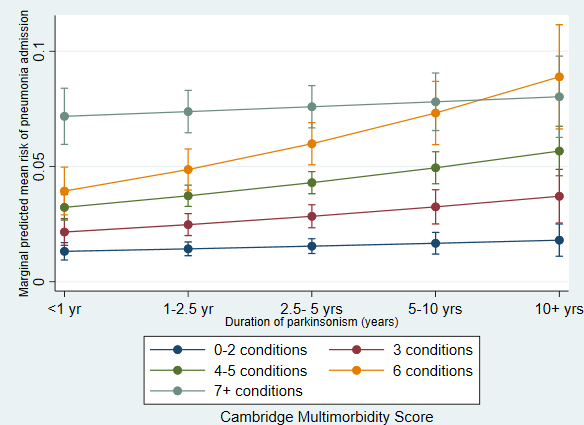


Figure S7: Predicted mean risk of emergency admission for pneumonia by multimorbidity score in patients according to duration of parkinsonism (years); p-value for likelihood ratio test = 0.029.


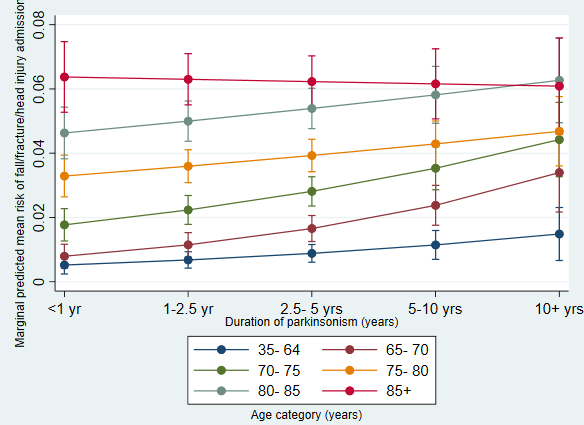


Figure S8: Predicted mean risk of admission for fall/fracture/head injury by age group in patients according to duration of parkinsonism (years); p-value for likelihood ratio test < 0.001.


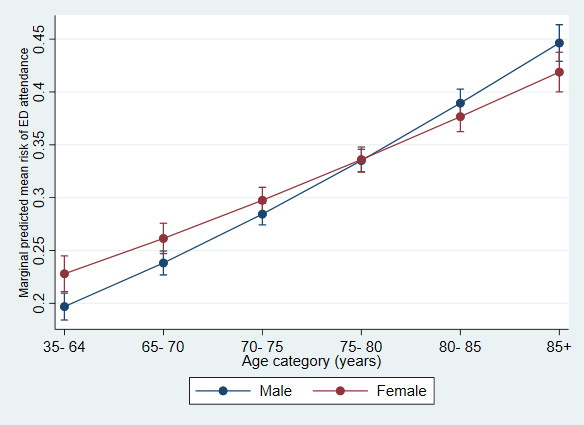


Figure S9: Predicted mean risk of emergency department attendance by age group (years) in men and women; p-value for likelihood ratio test = 0.001.


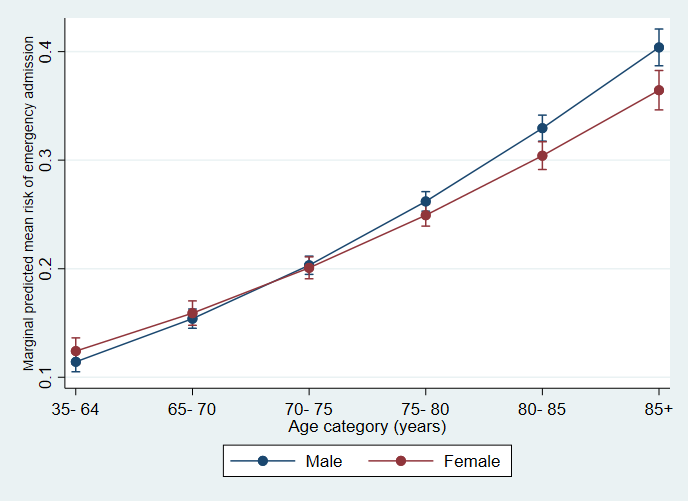


Figure S10: Predicted mean risk of emergency admission by age group (years) in men and women; p-value for likelihood ratio test = 0.010.


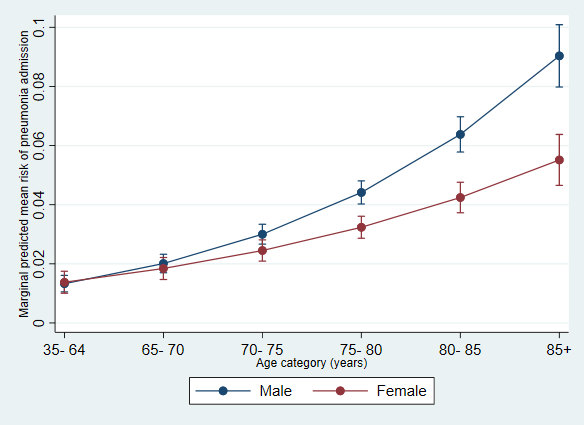


Figure S11: Predicted mean risk of emergency admission for pneumonia by age group (years) in men and women; p-value for likelihood ratio test = 0.010.

1. MHRA. Small area level data based on patient postcode: Documentation and Data Dictionary (set 22/January 2022) 2022 [Available from: <https://cprd.com/sites/default/files/2022-05/Documentation_SmallAreaData_Patient_set22_v3.3.pdf>.

2. MHRA. Small area level data based on practice postcode: Documentation and Data Dictionary 2022 [cited 2022 23rd November]. Available from: <https://cprd.com/sites/default/files/2022-05/Documentation_SmallAreaData_Practice_set22_v3.4_1.pdf>.

3. Gallagher AM, Dedman D, Padmanabhan S, Leufkens HGM, de Vries F. The accuracy of date of death recording in the Clinical Practice Research Datalink GOLD database in England compared with the Office for National Statistics death registrations. Pharmacoepidemiol Drug Saf. 2019;28(5):563-9

4. Textor J, van der Zander B, Gilthorpe MS, Liskiewicz M, Ellison GT. Robust causal inference using directed acyclic graphs: the R package 'dagitty'. Int J Epidemiol. 2016;45(6):1887-94
